# Data set on a study of gene expression in peripheral samples to identify biomarkers of severity of allergic and nonallergic asthma

**DOI:** 10.1016/j.dib.2016.12.035

**Published:** 2016-12-22

**Authors:** Selene Baos, David Calzada, Lucía Cremades, Joaquín Sastre, Joaquín Quiralte, Fernando Florido, Carlos Lahoz, Blanca Cárdaba

**Affiliations:** a Immunology Department, IIS- Jiménez Díaz Foundation, UAM, Madrid, Spain; b CIBERES, CIBER of Respiratory Diseases, Spain; c Allergy Department, Jiménez Díaz Foundation, Madrid, Spain; d Allergy Department, Vírgen del Rocío University Hospital, Seville, Spain; e Allergy Department, San Cecilio University Hospital, Granada, Spain

**Keywords:** Asthma, Allergy, Biomarkers, Gene expression, Peripheral samples

## Abstract

This article contains information related to the research article entitled “Biomarkers associated with disease severity in allergic and nonallergic asthma” (S. Baos, D. Calzada, L. Cremades, J. Sastre, J. Quiralte, F. Florido, C. Lahoz, B. Cárdaba, In press). Specifically, the clinical criteria stablished for selecting the study population (*n*=104 subjects) are described. Moreover, this article describes the criteria for selecting the 94 genes to be analyzed in PBMCs (peripheral blood mononuclear cells), it is provided a description of these genes and a Table with the genes most differentially expressed by clinical phenotypes and, finally it is detailed the experimental methodology followed for studying the protein expression of MSR1 (macrophage scavenger receptor 1), one of the genes evaluated in the research.

**Specifications Table**

**Table:** 

**Subject area**	Biology
**More specific subject area**	Immunology, biomarkers, asthma, allergy.
**Type of data**	Table, text file.
**How data was acquired**	Bibliographic search, qRT-PCR, Western Blot.
**Data format**	Raw
**Experimental factors**	Subjects´ diagnosis was done according to the GEMA (Spanish Guide for Asthma Management) classification. PBMCs were extracted from peripheral blood through gradient separation. RNA and protein from PBMCs were extracted with TRIzol´s method.
**Experimental features**	Genes selected were according to the 3 criteria stated. Through qRT-qPCR, gene expression differences among clinical groups were studied. The highest statistically significant data among the three clinical phenotypes are showed. Western blot was done to determine the protein expression of one of the genes studied.
**Data source location**	Madrid, Spain; Seville, Spain; Granada, Spain.
**Data accessibility**	Data is with this article.

**Value of the data**

• Data presented here shows the selection and clinical criteria [1] of the study population.
• Gene selection criteria of interesting candidates to be asthma´ biomarkers are provided in order to understand the validity of genes studied.
• A gene list of candidate biomarkers of asthma and allergy diseases is suggested for studying.
• A summary of the most differential genes among clinical phenotypes is showed. These data could be important for future biomarkers analyses.
• Western-blot method for MSR1 expression on protein extracted from PBMCs could be useful for future research.

## 1 Data

The data shown in the article give information on the criteria of patients´ selection and the criteria for choosing genes to be studied as candidate biomarkers for these diseases in peripheral samples. The specific western-blot method for the analysis of MSR1 expression on protein extracted from PBMCs is provided. Table 1 provides a list of candidate genes to be validated as relevant biomarkers and Table 2 summarize the possible biomarkers that differentiate asthmatic and allergic phenotypes.

## 2 Experimental design, materials and methods

### 2.1 Subjects

The study population comprised 104 unrelated subjects, 30 healthy control (HC) subjects, 30 patients with nonallergic asthma (NA), 30 with allergic asthma (AA), and 14 nonasthmatic allergic (AR) subjects. The samples of the groups with asthma came from the asthma biobank of the CIBERES (IIS-Fundación Jiménez Díaz-UAM, Madrid). A biorepository in which were included samples from clinically well-characterized subjects, from 5 Spanish Hospitals participant of this network (*Fundación Jiménez Díaz* Hospital and *Doce de Octubre* Hospital from Madrid, Doctor Negrín Hospital from Las Palmas de Gran Canaria, *Clinic* Hospital and *Sant* Pau Hospital both from Barcelona). These patients fulfilled the following criteria: severe, mild, or moderate asthma diagnosis assigned according to the GEMA [1]; no treatment was given before or during the collection of the samples. Pulmonary function test was determined by percentage of forced vital capacity (FVC) and forced vital volume in one second (FVE_1_). Patients with allergic asthma showed a positive skin prick test result for some of the airborne allergens from a battery of common allergens.

HCs were healthy subjects with no history of respiratory diseases. HCs and patients with allergy (rhinitis) without asthma were recruited and diagnosed at the Allergy Service of two hospitals in Andalusia (Spain), *Vírgen del Rocío* University Hospital from Seville, and *San Cecilio* University Hospital from Granada, Spain. AR patients fulfilled the following criteria: seasonal rhinitis without asthma, positive skin prick test for some of the airborne allergens from a battery of common allergens, and no previous immunotherapy.

HC and AR biological samples that were not used in this work were stored in the FJD Biobank, IIS-Fundación Jiménez Díaz Madrid.

Informed consent was obtained from each subject. Ethical approval for the study was obtained from the Ethical and Research Committee of the participating hospitals.

### 2.2 Gene selection criteria

Ninety-four genes (Table 1) were chosen following three main criteria for a gene expression analysis [2] through quantitative real time PCR with RNA of the study population described before:

a. Relevant genes associated with asthma and allergic diseases in more than one independent work, selected after a Pubmed literature search of analyses of differential gene expression, or polymorphic variants (SNPs) related to the disease.
b. Relevant genes previously described by our group [3].
c. Genes excluded by the other two criteria but that could be interesting due to their implication in cellular plasticity, inflammation, and/or regulation of the disease.

### 2.3 Gene expression analysis

Gene expression analysis between the 3 clinical groups is summarized in Table 2. The statistical analysis for testing differential gene expression was performed by using the StatMiner program (http://www.integromics.com/StatMiner). This program follows a simple, step-by-step analysis workflow guide that includes parametric, non-parametric, and paired tests for relative quantification of gene expression, as well as 2-way ANOVA for two-factor differential expression analysis. Significance was defined by RQ (relative quantification) <−2 or >2 and corrected *P* value (<0.05) adjusting the *P* value with the Benjamini–Hochberg FDR method.

### 2.4 MSR1 protein analysis by Western Blot

The protein expression of MSR1 was analyzed [2]. Specific protein was extracted from PBMCs (10^6^ cells) using the TRIzol method (Invitrogen, Carlsbad, CA, USA) and quantified by the BCA method (Thermo Scientific, Rockford, IL USA). Western blot used was the Invitrogen WesternBreeze^®^ Chemiluminescent Western Blot Immunodetection Kit (Life Technologies) following the manufacturer׳s instructions with minor modifications. Briefly, 40 μg of proteins from each subject were running in a 12% SDS-PAGE Novex Bolt™ Mini gels (Life Technologies) and transferred using the Invitrogen Blot^®^ Dry Blotting System to nitrocellulose membranes. After 30 min of incubation with blocking solution, were incubated overnight at 4 °C with rabbit anti-human polyclonal CD204/Macrophage Scavenger Receptor I antibody (dilution 1:2500) (Thermo Scientific) as specific antibody and, with a rabbit anti-human monoclonal β-Actin antibody (dilution 1:1000) (Cell Signalling Technology, Danvers, MA, USA) as control. The result was visualized by chemiluminiscence using a luminescent image analyzer: ImageQuant LAS 4000 (GE Healthcare Life Science, Little Chalfont, Buckinghamshire, UK). Data of MSR1 results were relativize to β-Actin expression.

## Funding sources

This work was supported in part by research grants PI13/01730 co-supported by FEDER, CIBERES (ISCIII, 0013) and Biobank (PT13/0010/0012) from the Fund for Health Research (Spanish Ministry of Economy and Competitiveness). S. Baos was supported by CIBERES (ISCIII, 0013) and Conchita Rábago Foundation. D. Calzada by *Conchita Rábago* Foundation, Madrid, Spain. L. Cremades was supported by a contract from MINECO (PEJ-2014-A-31609, *Sistema de Garantía Juvenil*), cofinanced by European Social Fund (ESF) and Youth Employment Initiative (YEI).

## Figures and Tables

**Table 1:** List of the 94 genes studied.

**Gene symbol**	**Gene name**	**Selection criteria**	**Detector**
***ADAM33***	*ADAM metallopeptidase domain 33*	3	ADAM33-Hs00905552_m1
***ADRB1***	*adrenoceptor beta 1*	2	ADRB1-Hs02330048_s1
**AKT1**	*v-akt murine thymoma viral oncogene homolog 1*	2	AKT1-Hs00178289_m1
***ALOX15***	*arachidonate 15-lipoxygenase*	1	ALOX15-Hs00993765_g1
***ALOX5***	*arachidonate 5-lipoxygenase*	2	ALOX5-Hs01095330_m1
***APAF1***	*apoptotic peptidase activating factor 1*	2	APAF1-Hs00559441_m1
***BAX***	*BCL2-associated X protein*	2	BAX-Hs00180269_m1
***C3AR1***	*complement component 3a receptor 1*	2	C3AR1-Hs00269693
***CCL11***	*chemokine (C-C motif) ligand 11*	3	CCL11-Hs00237013_m1
***CCL-17***	*chemokine (C-C motif) ligand 17*	3	CCL17-Hs00171074_m1
**CCL5**	*chemokine (C-C motif) ligand 5*	3	CCL5-Hs00982282_m1
**CD40**	*CD40 molecule, TNF receptor superfamily member 5*	2	CD40-Hs01002913_g1
**CD48**	*CD48 molecule*	2	CD48-Hs00914738_m1
**CD86**	*CD86 molecule*	2	CD86-Hs01567026_m1
***CHI3L1***	*chitinase 3-like 1 (cartilage glycoprotein-39)*	1	CHI3L1-Hs00609691_m1
***CLCA1***	*chloride channel accessory 1*	1	CLCA1-Hs00976287_m1
**CPA3**	*carboxypeptidase A3 (mast cell)*	1	CPA3-Hs00157019_m1
***CRTAP***	*cartilage associated protein*	2	CRTAP-Hs00197261_m1
***CTSC***	*cathepsin C*	1	CTSC-Hs00175188_m1
***CTSG***	*cathepsin G*	1	CTSG-Hs01113415_g1
***CX3CR1***	*chemokine (C-X3-C motif) receptor 1*	1	CX3CR1-Hs01922583_s1
***DUSP1***	*dual specificity phosphatase 1*	1	DUSP1-Hs00610256_g1
***RNASE3***	*ribonuclease, RNase A family, 3*	3	RNASE3-Hs01923184_s1
***EIF5A***	*eukaryotic translation initiation factor 5A*	1	EIF5A-Hs00744729_s1
***FOXP3***	*forkhead box P3*	3	FOXP3-Hs01085834_m1
**FPR3**	*formyl peptide receptor 3*	2	FPR3-Hs00266666_s1
***GADD45B***	*growth arrest and DNA-damage-inducible, beta*	1	GADD45B-Hs04188837_g1
**GPX3**	*glutathione peroxidase 3 (plasma)*	1	GPX3-Hs01078668_m1
***GZMH***	*granzyme H (cathepsin G-like 2, protein h-CCPX)*	2	GZMH-Hs00277212_m1
***HLA-DQB1***	*major histocompatibility complex, class II, DQ beta 1*	1, 2	HLA-DQB1-Hs03054971_m1
***HLA-DRB1***	*major histocompatibility complex, class II, DR beta 1*	2	HLA-DRB1-Hs99999917_m1
***IFNG***	*interferon, gamma*	3	IFNG-Hs00989291_m1
***IL-10***	*interleukin 10*	2	IL10-Hs00961622_m1
***IL13***	*interleukin 13*	1	IL13-Hs00174379_m1
***IL-17***	*interleukin 17A*	3	IL17A-Hs00174383_m1
***IL1R1***	*interleukin 1 receptor, type I*	1	IL1R1-Hs00991002_m1
***IL1R2***	*interleukin 1 receptor, type II*	1	IL1R2-Hs01030384_m1
***IL-2***	*interleukin 2*	3	IL2-Hs00174114_m1
***IL-25***	*interleukin 25*	3	IL25-Hs03044841_m1
***IL2RB***	*interleukin 2 receptor, beta*	1	IL2RB-Hs01081697_m1
**IL33**	*interleukin 33*	1	IL33-Hs00369211_m1
***IL-4***	*interleukin 4*	3	IL4-Hs00174122_m1
**IL4R**	*interleukin 4 receptor*	3	IL4R-Hs00166237_m1
***IL5***	*interleukin 5*	1	IL5-Hs01548712_g1
***IL6***	*interleukin 6 (interferon, beta 2)*	1, 2	IL6-Hs00985639_m1
***IL8***	*interleukin 8*	1	IL8-Hs00174103_m1
***IL-9***	*interleukin 9*	3	IL9-Hs00914237_m1
***IRAK3***	*interleukin-1 receptor-associated kinase 3*	3	IRAK3-Hs00936103_m1
***ITGAL***	*integrin, alpha L (antigen CD11A (p180), lymphocyte function-associated antigen 1; alpha polypeptide)*	2	ITGAL-Hs00158218_m1
***ITGB7***	*integrin, beta 7*	2	ITGB7-Hs01565750_m1
***ITGB8***	*integrin, beta 8*	2	ITGB8-Hs00174456_m1
***LCK***	*lymphocyte-specific protein tyrosine kinasep*	2	LCK-Hs00178427_m1
***LGALS3***	*lectin, galactoside-binding, soluble, 3*	3	LGALS3-Hs00173587_m1
***LYN***	*v-yes-1 Yamaguchi sarcoma viral related oncogene homolog*	2	LYN-Hs00176719_m1
***MAPK13***	*mitogen-activated protein kinase 13*	2	MAPK13-Hs00559623_m1
**MSR1**	*macrophage scavenger receptor 1*	2	MSR1-Hs00234007_m1
**MUC2**	*mucin 2, oligomeric mucus/gel-forming*	1	MUC2-Hs03005103_g1
***MUC5AC***	*mucin 5AC, oligomeric mucus/gel-forming*	1	MUC5AC-Hs00873651_Mh
***MUC5B***	*mucin 5B, oligomeric mucus/gel-forming*	1	MUC5B-Hs00861595_m1
**NCF2**	*neutrophil cytosolic factor 2*	1	NCF2-Hs01084940_m1
***NFATC1***	*nuclear factor of activated T-cells, cytoplasmic, calcineurin-dependent 1*	2	NFATC1-Hs00542678_m1
***NFKBIZ***	*nuclear factor of kappa light polypeptide gene enhancer in B-cells inhibitor, zeta*	1	NFKBIZ-Hs00230071_m1
***NLRP3***	*NLR family, pyrin domain containing 3*	2	NLRP3-Hs00918082_m1
***NOS2A***	*nitric oxide synthase 2, inducible*	1	NOS2-Hs01075529_m1
***ORMDL3***	*ORM1-like 3 (S. cerevisiae)*	1	ORMDL3-Hs00918021_m1
***PHLDA1***	*pleckstrin homology-like domain, family A, member 1*	1	PHLDA1-Hs00705810_s1
***PI3***	*peptidase inhibitor 3, skin-derived*	1	PI3-Hs00160066_m1
***POSTN***	*periostin, osteoblast specific factor*	1	POSTN-Hs01566734_m1
***PRKACA***	*protein kinase, cAMP-dependent, catalytic, alpha*	2	PRKACA-Hs00427274_m1
***PRKACB***	*protein kinase, cAMP-dependent, catalytic, beta*	2	PRKACB-Hs01086757_m1
***PTGER2***	*prostaglandin E receptor 2 (subtype EP2*), 53 *kDa*	2	PTGER2-Hs04183523_m1
***PTPRC***	*protein tyrosine phosphatase, receptor type, C*	3	PTPRC-Hs04189704_m1
***S100A9***	*S100 calcium binding protein A9*	1	S100A9-Hs00610058_m1
***S1PR5***	*sphingosine-1-phosphate receptor 5*	2	S1PR5-Hs00928195_s1
***SCD***	*stearoyl-CoA desaturase (delta-9-desaturase)*	1	SCD-Hs01682761_m1
***SELL***	*selectin L*	2	SELL-Hs00174151_m1
***SERPINB2***	*serpin peptidase inhibitor, clade B (ovalbumin), member 2*	1, 2	SERPINB2-Hs01010736_m1
***SERPINB4***	*serpin peptidase inhibitor, clade B (ovalbumin), member 4*	1	SERPINB4-Hs01691258_g1
***SMURF1***	*SMAD specific E3 ubiquitin protein ligase 1*	2	SMURF1-Hs00905759_m1
**SOS1**	*son of sevenless homolog 1 (Drosophila)*	2	SOS1-Hs00362308_m1
***SPN***	*sialophorin*	2	SPN-Hs01872322_s1
**SPP1**	*secreted phosphoprotein 1*	3	SPP1-Hs00959010_m1
***STAT1***	*signal transducer and activator of transcription* 1.91 *kDa*	3	STAT1-Hs01013996_m1
***SVIL***	*supervillin*	1	SVIL-Hs00931028_m1
***TAGAP***	*T-cell activation RhoGTPase activating protein*	2	TAGAP-Hs00299284_m1
***TCF21***	*transcription factor 21*	1	TCF21-Hs00162646_m1
***TGFB1***	*transforming growth factor, beta 1*	2	TGFB1-Hs00998133_m1
**TLR4**	*toll-like receptor 4*	2	TLR4-Hs00152939_m1
***TNFA***	*tumor necrosis factor*	3	TNF-Hs01113624_g1
***TNFAIP3***	*tumor necrosis factor, alpha-induced protein 3*	1	TNFAIP3-Hs00234713_m1
***TRIM37***	*tripartite motif containing 37*	2	TRIM37-Hs00248701_m1
***TSLP***	*thymic stromal lymphopoietin*	1	TSLP-Hs00263639_m1
***VCAN***	*versican*	2	VCAN-Hs00171642_m1
***ZAP70***	*zeta-chain (TCR) associated protein kinase* 70 *kDa*	2	ZAP70-Hs00896347_m1

Selection criteria were: 1. Relevant genes by differential expression or SNP studies in asthma/allergy, which were found in more than one independent work following a literature search; 2. Genes with differential expression found in results of previous studies from our laboratory; 3. Genes of interest because of their role in cellular plasticity, inflammation and/or regulation that could have been excluded by the other criteria. The detector refers to the specific primer of each gene used to carry out qRT-PCR.

**Table 2:** Differential genes among clinical phenotypes.

**Comparison**	**Number of genes with significant differential expression**⁎	**Number of genes upregulated**	**Number of genes downregulated**	**Genes statistically significant with a RQ>10**
**NA*****vs*****AA**	74	74 in NA	–	*CCL5, CHI3L1, CTSG, GMH, IL1-R2*
**NA*****vs*****AR**	66	64 in NA	2 in NA	*CCL5, CRTAP, GPX3,*
				*HLA-DQB1, IL-10, IL2RB, MSR1, NLRP3, PHLDA1, SERPINB2, **PI3***
**AA*****vs*****AR**	14	4 in AA	10 in AA	***CHI3L1, CPA3, CTSG, PI3***

NA: Nonallergic asthma group; AA: allergic asthma group; AR: nonasthmatic allergy (rhinitic) group; RQ: relative quantification.

⁎ Significance established at an adjusted *p*<0.05 and a RQ <−2 or >2. All genes mentioned in the last column are overexpressed except the ones marked in bold which are underexpressed.
